# Precipitation Regime Shift Enhanced the Rain Pulse Effect on Soil Respiration in a Semi-Arid Steppe

**DOI:** 10.1371/journal.pone.0104217

**Published:** 2014-08-05

**Authors:** Liming Yan, Shiping Chen, Jianyang Xia, Yiqi Luo

**Affiliations:** 1 State Key Laboratory of Vegetation and Environmental Change, Institute of Botany, Chinese Academy of Sciences, Beijing, China; 2 School of Life Sciences, Fudan University, Shanghai, China; 3 Department of Microbiology and Botany, University of Oklahoma, Norman, Oklahoma, United States of America; National University of Mongolia, Mongolia

## Abstract

The effect of resource pulses, such as rainfall events, on soil respiration plays an important role in controlling grassland carbon balance, but how shifts in long-term precipitation regime regulate rain pulse effect on soil respiration is still unclear. We first quantified the influence of rainfall event on soil respiration based on a two-year (2006 and 2009) continuously measured soil respiration data set in a temperate steppe in northern China. In 2006 and 2009, soil carbon release induced by rainfall events contributed about 44.5% (83.3 g C m^−2^) and 39.6% (61.7 g C m^−2^) to the growing-season total soil respiration, respectively. The pulse effect of rainfall event on soil respiration can be accurately predicted by a water status index (WSI), which is the product of rainfall event size and the ratio between antecedent soil temperature to moisture at the depth of 10 cm (*r* ^2^ = 0.92, *P*<0.001) through the growing season. It indicates the pulse effect can be enhanced by not only larger individual rainfall event, but also higher soil temperature/moisture ratio which is usually associated with longer dry spells. We then analyzed a long-term (1953–2009) precipitation record in the experimental area. We found both the extreme heavy rainfall events (>40 mm per event) and the long dry-spells (>5 days) during the growing seasons increased from 1953–2009. It suggests the shift in precipitation regime has increased the contribution of rain pulse effect to growing-season total soil respiration in this region. These findings highlight the importance of incorporating precipitation regime shift and its impacts on the rain pulse effect into the future predictions of grassland carbon cycle under climate change.

## Introduction

Global precipitation has been predicted to change with increasing intra-annual variability and more frequent extreme rainfall events [1], [2]. Such shifts in precipitation regime could have profound impacts on belowground carbon (C) release, especially in the arid and semiarid ecosystems [3]–[6]. It has been widely reported that soil respiration in grassland ecosystems can increase significantly and immediately after a rainfall event, followed by a decrease with declining soil moisture [4], [7]. This pulse effect of rainfall event on soil respiration has been suggested as an important contributor to ecosystem C release in grassland ecosystems [3], [8], [9]. Therefore, a better understanding of how the changing precipitation regime will affect rain pulse effect is important for predicting future grassland C feedbacks to climate change.

Previous studies have summarized that variations in soil respiration are determined by several factors, including soil temperature, soil water availability and carbon substrate supply [10]–[12]. Yet it is not clear which factors control the pulse effect of a single rainfall event on soil respiration. There have been both laboratory [13]–[15] and field [7], [16], [17] attempts trying to identify the regulatory mechanisms of rain pulses on soil respiration. The magnitude of soil respiration response (e.g., soil carbon release) is positively correlated with rainfall event size [4], [18], [19]. Other studies (e.g. [20]) have observed that the antecedent soil water condition is also an important influencing factor. The pulse effect is intensified by the dry condition of the antecedent soil [20], and the effect is less obvious if the soil is wet before the rainfall event [21]–[23]. Soil temperature has been widely reported to regulate soil microbial activities and thus soil heterotrophic respiration in various ecosystems [22], [24]. Other conditions, such as plant activity [25], [26] and soil organic matter content [20], [27], are also believed to influence the effects of rainfall event on soil respiration. Therefore, the predictability of rain pulse effect on soil respiration is still low and no effective approach or indicator has been developed so far.

In semi-arid ecosystems, water availability is the dominant factor regulating soil respiration [28]–[30]. Water availability also moderates the effects of the other factors on soil respiration such as temperature and substrate supply [30]. Water availability and its intra- and inter-annual variations are directly linked to both the intensity and the frequency of precipitation. Although IPCC [2] has reported a trend of significant change in both total amount and temporal patterns of precipitation, only a few studies have analyzed the general changes of precipitation regimes especially in semi-arid temperate grasslands [31]. Shifts in precipitation regime will alter not only the size of individual rainfall event, but also the length of the dry-spell duration and thus the antecedent soil water condition. Previous laboratory experiments have shown that both of these two factors can significantly influence the soil CO_2_ release [32], [33]. A growing body of works using field experiments have demonstrated that shifts in precipitation regime alone, even when the total rainfall amount does not change, can have large impacts on grassland soil CO_2_ release [29], [34], [35] and its pulse responses to rainfall events [4], [17]. However, to our knowledge, few studies have observed and quantified the dynamics of soil respiration after rainfall events [36], [37]. Therefore, it is necessary to incorporate the changes in precipitation regime when evaluating the effect of rainfall event on C cycling in natural grassland ecosystems.

The area of grasslands contributed to about 40% of the total territory of China [38], and the temperate steppe in China is the third largest grassland area in the world [39]. In this study, we analyzed both a long-term (1953–2009) rainfall data set and two years (2006 and 2009) of continuously measured soil respiration data in a temperate steppe in northern China. We attempt to address the following questions: (1) What are the controlling factors of the rainfall event effect on soil respiration through the growing season in this ecosystem? (2) Has precipitation regime changed over the last ∼60 yr in this semi-arid steppe? (3) How shifting precipitation regime would influence soil respiration responses to pulses in water availability?

## Materials and Methods

### 2.1. Site description

Our field site (42°27′N, 116° 41′E), located Duolun County in the northeastern Inner Mongolia, China, belongs to Duolun Restoration Ecology Experimentation and Demonstration Station (DREEDS), Institute of Botany. No specific permissions were required for scientific researches. The site has been fenced in to exclude grazing since 2001, and no other management like mowing or fertilizing was applied. The mean altitude is about 1430 m above sea level. The vegetation was dominated by C3 grasses (e.g. *Stipa krylovii, Agropyron cristatum, Leymus chinensis*) and a semi-shrub species (*Artemisia frigida*). The soil in our study site is classified as Calcic-orthic Aridisol. The mean annual air temperature is 3.3°C and the mean annual precipitation is 377 mm, 95% (i.e. 358 mm) of which occurs during the growing season from April to October. Average air temperature was 3.13°C in 2006 and 3.09°C in 2009. Annual precipitation was 425 mm in 2006 and 185 mm in 2009, respectively.

### 2.2 Variable measurement

The growing season in this ecosystem usually starts from late April and ends in early October. Five PVC collars (20.3 cm in diameter and 8 cm in height) were inserted into the soil to a depth of 3 cm. The PVC collars were randomly distributed in the study site with 5 m–30 m distance between any two of them in late April, 2006, and covered an area of about 300 m^2^. The site is flat so topography had little impact on the difference in measured soil respiration among replicates. Soil respiration rates were continuously measured during the growing season (from May to October) of 2006 and 2009 on a half-hour basis using an infra-red gas analyzer (LI-840, Li-Cor Inc., Lincoln, NE, USA) that was connected to five automatic measurement chambers. All living plants inside the chambers were clipped by hand weekly to exclude aboveground plant respiration. The clipped aboveground plants were left in the chambers to include CO_2_ efflux from the litter decomposition. The CO_2_ concentrations in the chambers were recorded in CR1000 data logger (Campbell Scientific Inc., CSI, Utah, USA), and were processed by LoggerNet 3.1 (CSI, USA). Each measurement took 120 s. The soil respiration rates were determined from the time series of soil CO_2_ efflux concentrations. Since the measuring system worked well during 2006 and 2009, only small data gaps (less than 2 h) existed in two years data sets and were filled by linear interpolation method.

Soil temperatures (°C) at a depth of 10 cm and volumetric soil moisture (%) at a depth of 0–10 cm were measured using 107 soil temperature probes (CSI, USA) and CS616 soil water probes (CSI, USA), respectively. Three combinations of soil temperature and water probes were placed close (about 1 m) to the soil respiration chambers. The mean values of half-hour soil temperature and moisture were recorded in a CR1000 data logger (CSI, USA) simultaneously.

The precipitation data of the past 57 years were provided by local meteorological stations in Duolun County. The precipitation data of the growing season of 2006 and 2009 were measured by a tipping bucket rain gauge (TE525MM, CSI) at the study site.

### 2.3. Statistical analyses

Considering the significant impact of soil temperature on the diurnal variation of soil respiration, we analyzed precipitation and soil respiration data on a daily time step. There was a rainfall event when precipitation was recorded, and a day without any precipitation was defined as a dry day. The rainfall event size was defined as the total amount of rainfall during a rainfall event. Note that one day may have more than one rainfall event and, at the other extreme, one rainfall event may last for a few days. An uninterrupted sequence of dry days preceded and succeeded by at least one rainfall event is referred to as a dry-spell event [40], [41]. We added up the total precipitation amount and calculated the frequencies of different dry-spell durations (days) in growing seasons of the past 57 years (1953–2009). The rainfall and dry-spell events were then classified into different categories based on their size (rainfall event size: 0–2, 2–5, 5–10, 10–15, 15–20, 20–30, 30–40, 40–50, or >50 mm) and duration (dry-spell duration: 0–5, 5–10, 10–20, 20–30, or >30 days).

Since the size of individual rainfall events varies greatly, the rain pulse effect on soil respiration can last from several hours to a few weeks. Thus, if the rain pulse response lasted for at least three days, we would use daily soil respiration data with the following function to estimate the effect of a rainfall event on soil respiration [7]:
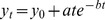
(1)where *y_t_* is daily soil respiration rate after the rainfall event, *y_0_* is initial daily soil respiration before the rainfall event, *t* is the time (continuous but not a discrete daily time-step) after the rainfall event, and *a* and *b* are coefficients. However, if the rain pulse response lasted less than three days, we would compare the difference between post-rainfall soil respiration and the control at half-hourly time scale. The sum of all the differences over one day greater than 0±0.01 (g C m^−2^ day^−1^) was defined as rainfall event. The calculation was applied for each chamber. Based on the measurements, there were totally 47 and 45 rainfall events during the growing season of 2006 and 2009, respectively. According to their durations, the half-hourly analyses were applied to 10 and 13 rainfall events in 2006 and 2009, respectively. We further defined the time for the soil respiration response to peak after a rainfall event as the peak time (T_peak_), and calculated the duration of the pulse (T_duration_) before soil respiration decreased to 99% of the antecedent soil respiration. The rain pulse effects were estimated by the accumulative differences during this period. These analyses were conducted using Matlab (Mathworks, Natick, MA).

Linear regression analyses were used to evaluate the relationships between rain pulse response patterns (T_peak_, T_duration_, pulse effect) of soil respiration, rain size and water status index (WSI) in the growing seasons of 2006 and 2009. The water status index (WSI) was defined as,
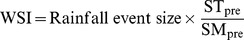
(2)where ST_pre_ and SM_pre_ are the soil temperature and moisture at 10 cm depth before a rainfall event. The index thus takes both rainfall event size and antecedent condition into consideration. The development of the WSI was based on the findings from previous studies, which have shown that larger rainfall event size [4] as well as drier [23] and warmer [22] pre-event soil conditions positively affect rain pulse effect. Growing-season total soil respiration was defined as the sum of daily soil respiration from May to October in each year.

## Results

### 3.1. Growing-season total soil respiration and relative environmental factors

**Figure 1:**
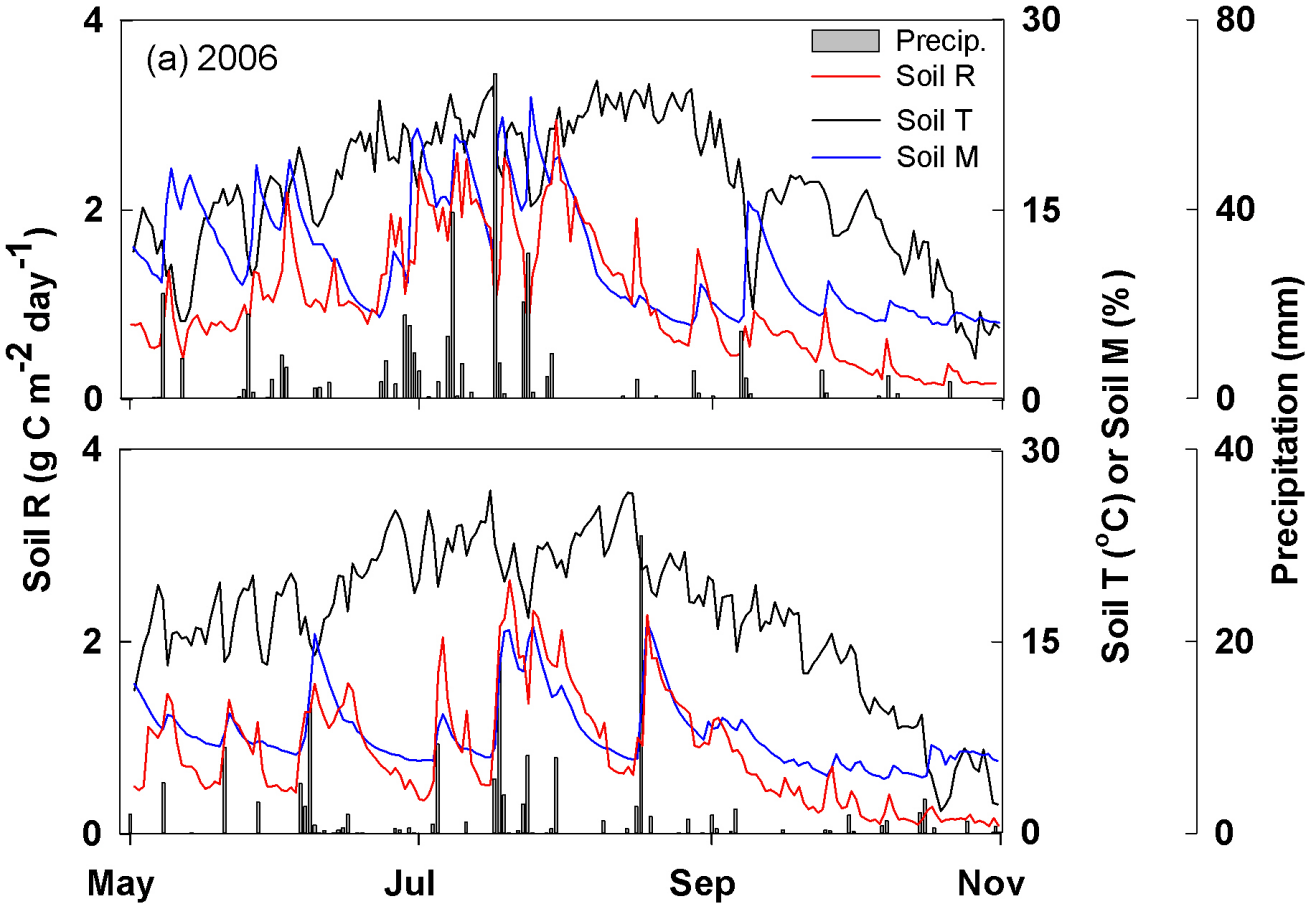
Seasonal patterns of precipitation (gray rectangle), soil moisture (black dashed line), soil temperature (black line) and soil respiration (gray line) during the growing seasons of 2006 (a) and 2009 (b).

Soil respiration rates showed clear seasonal variations and reached maximum values in the middle of the growing seasons in both 2006 and 2009 (Fig. 1), with the growing-season total soil respiration as 187.2±16.47 g C m^−2^ in 2006 and 155.9±4.58 g C m^−2^ in 2009. Precipitation during the growing season (May to October) was slightly above the long-term average in 2006 (403 mm) but was far below the average in 2009 (168 mm) (Fig. 1). In both years, the heaviest rainfall occurred in the middle of the growing season (late July to early August; Fig 1). Accordingly, soil moisture fluctuated through the growing season in both years, with the highest soil moisture occurred in late July in both years (Fig. 1). Soil temperature had a pronounced seasonal dynamic in both years, with the highest temperature occurring in late July of 2006 and early August of 2009 (Fig. 1). Lower seasonal mean soil temperature (16.86°C) and higher soil moisture (11.26%) were found in 2006 than those of 2009 (17.14°C and 7.81%).

### 3.2. Pulse effects of soil respiration and its controlling factors

**Figure 2:**
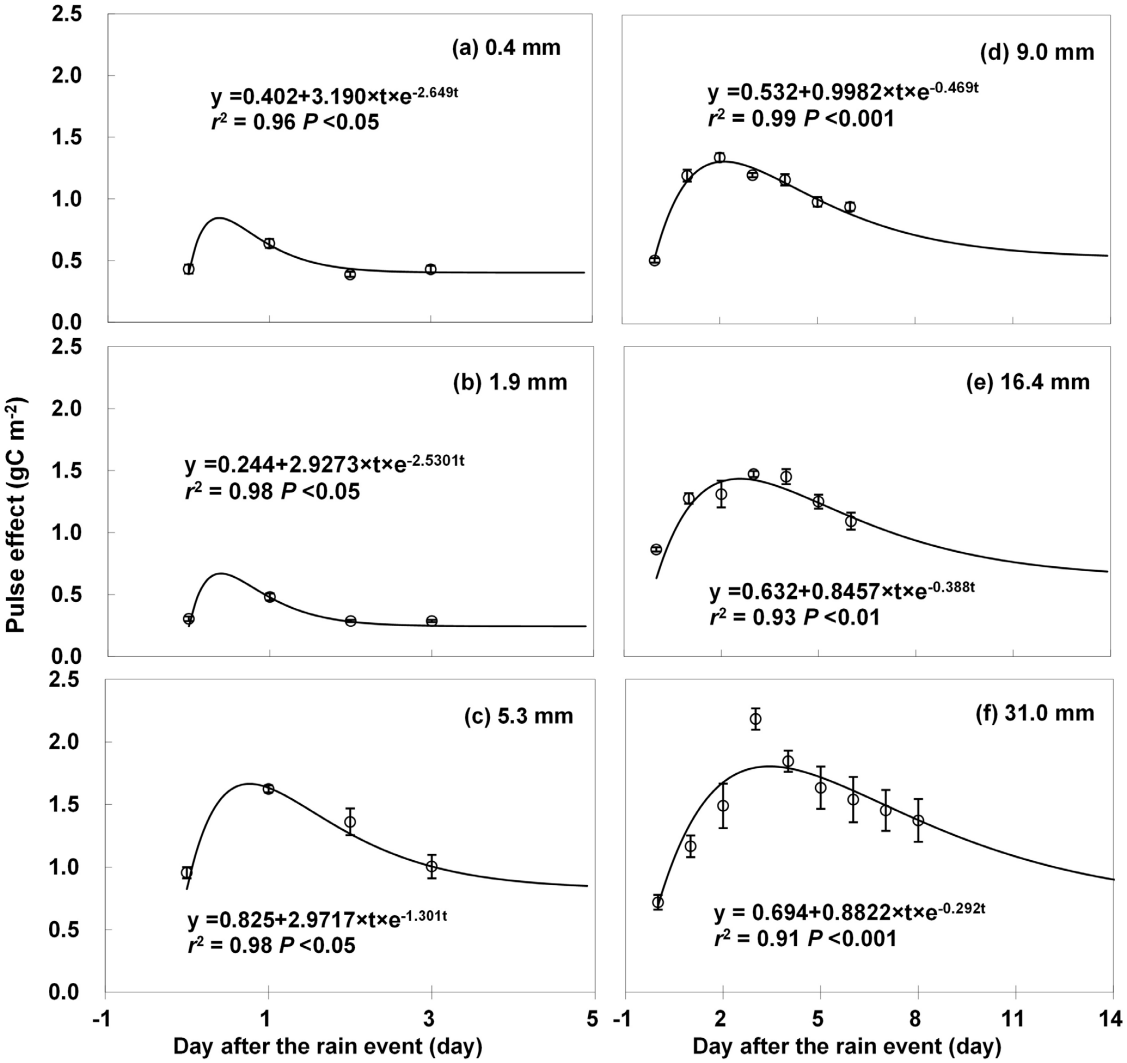
The response of soil respiration to rain pulses under different rain sizes. Open circles are the measured data and shown as mean ± standard deviation. Exponential equations in the form of *y* = *y_0_*+*at*e^−*b*t^ are used to fit the data (represented by solid lines). Rain size is shown in each panel. The 0 in the x-axis represents the day before the rainfall event.

**Figure 3:**
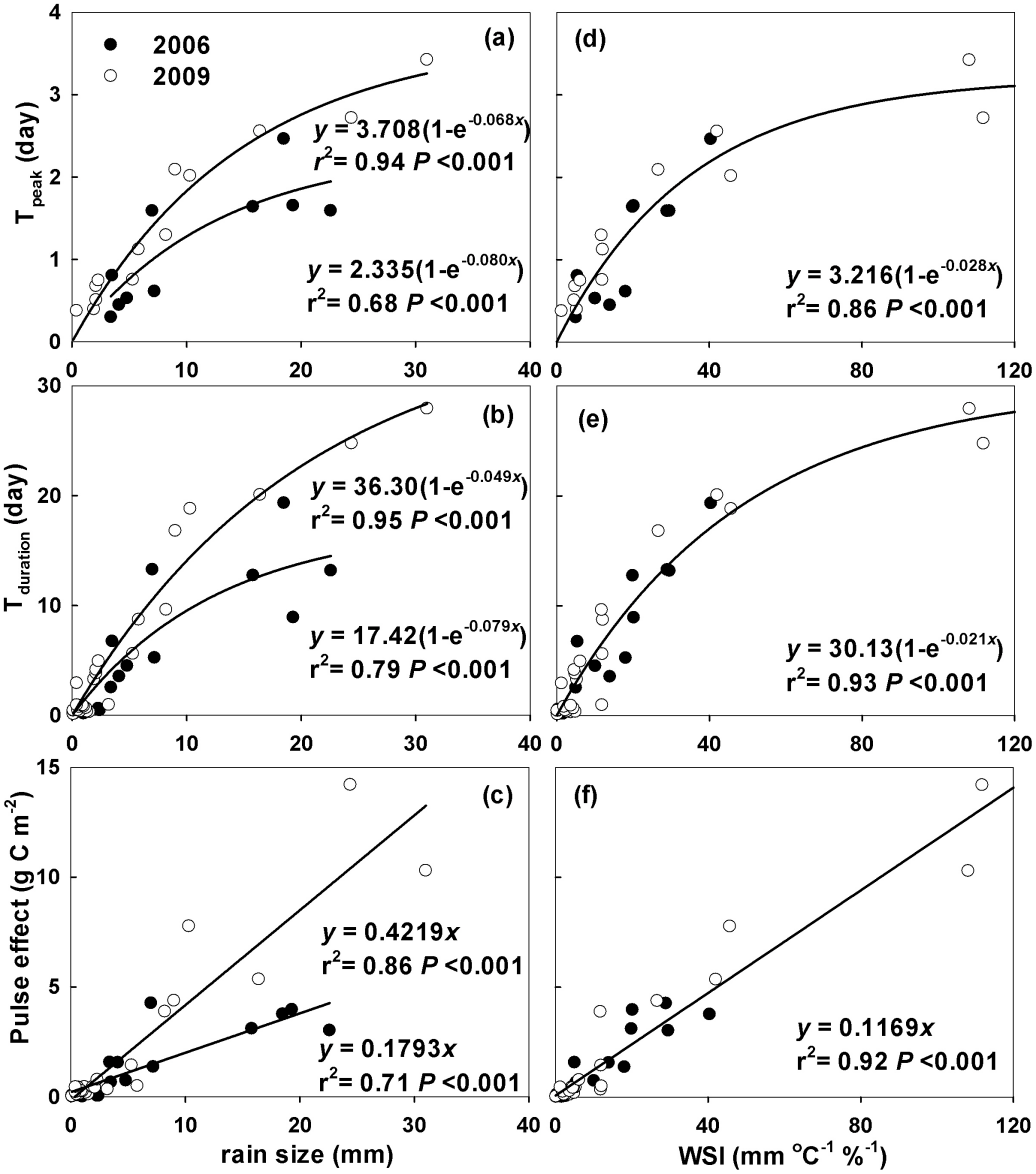
The relationship between dependent variables including the peak time (T_peak_) and the duration (T_duration_) of the pulses, and the pulse effects on soil respiration, and independent variables including rain size (a, b, c) and WSI (rain size × the ratio of antecedent soil temperature to soil moisture; d, e, f) in 2006 (solid circle) and 2009 (open circle), respectively.

Equation (1) well fitted the temporal patterns of soil respiration following heavy rainfall events, with *r* ^2^ ranging from 0.91 to 0.99 (*P*<0.05; Fig. 2a–f). In 2009, for example, daily soil respiration rates increased by 72–160% within 2 days following rainfall events (Fig. 2d–f). For small rainfall events, daily average of soil respiration could not capture the rapid responses of soil respiration to rainfall events. For example, similar to the predicted curves in Figure 3, the rainfall event effect on soil respiration peaked within several hours of the end of small rainfall events (Figure S1 in File S1). After reaching the peak, the soil respiration rate gradually returned to its antecedent level in 2.93 to 27.9 days after rainfall events of 0.4 mm to 31 mm, respectively (estimated from the fitted curve in Fig. 2). Both the magnitude and duration of the pulse effect on soil respiration (T_peak_ and T_duration_) were positively correlated with rainfall event size (Fig. 3a–c). A higher sensitivity of rainfall event effect to rainfall event size was found in 2009 than in 2006 (Fig. 3c). It could be ascribed to the higher sensitivities of T_peak_ and T_duration_ to rainfall event size in 2009 than in 2006 (Fig. 3a, b). It suggested that rainfall event size alone was not a good indicator for the effect of rainfall event on soil respiration in this ecosystem. When compared against WSI instead of rainfall event size, however, there were no significant differences in regressive curves between 2006 and 2009 (*P*>0.1) (Fig. 3d–f). The values of T_peak_ (about 3 days; *r* ^2^ = 0.86, *P*<0.001) and T_duration_ (about 30 days; *r* ^2^ = 0.94, *P*<0.001) of the pulse responses increased logarithmically with the WSI (Fig. 3d and e). Accumulative soil respiration during the pulse processes increased linearly with the WSI (Pulse effect  = 0.1169×WSI, *r* ^2^ = 0.92, *P*<0.001, Fig. 3f). Based on this relationship, we calculated the pulse effect of each rainfall event on soil respiration, and found the pulse effect contributed 44.5% and 39.6% of the measured growing-season total soil respiration in 2006 and 2009, respectively.

### 3.3. Long-term trend of the precipitation regime during 1953-2009

**Figure 4:**
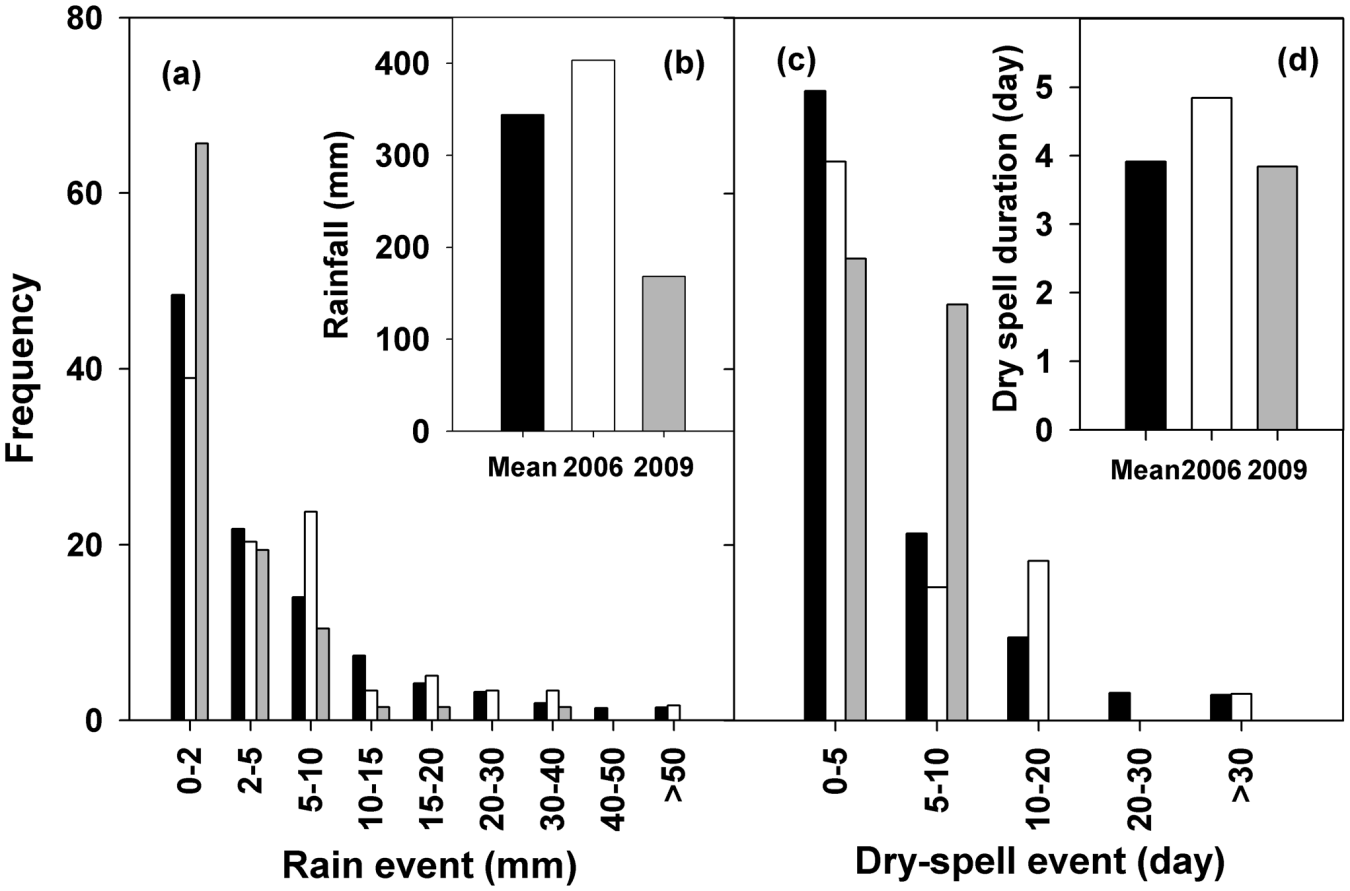
The frequency distribution of different class-categories of rainfall events (a) and dry-spell events (c) during the growing seasons of 2006 and 2009. The long-term mean annual precipitations from 1953 to 2009 are also represented here by black columns. The insert panels show the precipitation during the growing season (b) and the mean dry-spell duration during the growing season (d) in 2006 and 2009.

The mean precipitation during the growing season from 1953-2009 was 358 mm (Fig. 4b). The frequency of rainfall event occurrence decreased with increasing amount of rainfall (Fig. 4a). Long-term mean rainfall frequency with rainfall amount of 0–2 mm, 2–5 mm, 5–10 mm, 10–15 mm, 15–20 mm, 20–30 mm, 30–40 mm, 40–50 mm, and >50 mm per event was 48.4%, 21.8%, 14.0%, 7.3%, 3.8%, 2.8%, 1.0%, 0.4%, and 0.4%, respectively (Fig. 4a). We classified the duration of dry-spell into five groups (<5 days, 5–10 days, 10–20 days, 20–30 days, and >30 days). The long-term mean occurrence frequency of the dry-spells was 71.7%, 21.4%, 5.9%, 0.8%, and 0.3% for the five groups, respectively (Fig. 4c).

During the 57 years, no clear temporal trend of the total growing-season precipitation was found (Figure S2 in File S1). The 40–50 mm (*r* ^2^ = 0.16, *P* = 0.061) and >50 mm (*r* ^2^ = 0.23, *P*<0.05) rainfall events showed significant increasing tendencies over the years (Fig. S2k and l in File S1), while those of the smaller-sized rainfall events did not show any clear trend (Figure S2b–g in File S1). The mean dry-spell duration of the growing season increased from 1953 to 2009 (*P*<0.001; Figure S3a). During these 57 years, the frequency of dry spell events <5 days in duration significantly decreased (*r* ^2^ = 0.28, *P*<0.001; Figure S3b in File S1), while the dry-spell events of 5–10 days (*r* ^2^ = 0.13, *P* = 0.003) and 10–20 days (*r* ^2^ = 0.11, *P* = 0.01) occurred more frequently (Figure S3c–d in File S1). The frequency of dry-spell events longer than 20 days did not change during this time period (Figure S3e–f in File S1).

## Discussion

### 4.1. Factors controlling rain pulse on soil respiration

In this study, significant pulse responses of soil respiration were found after rainfall events (Fig. 2), which was consistent with the results reported in other arid ecosystems [18], [25]. The rain pulse induced soil respiration was 44.5% (83.3 g C m^−2^) and 39.6% (61.7 g C m^−2^) of the growing-season total soil respiration in 2006 and 2009, respectively. Since only few studies have quantified the contribution of rain pulse effect to growing-season total soil respiration, it is difficult to compare our results to those from other grasslands. However, some evidence show that the contribution of rain pulse to total soil respiration is lower in forest ecosystems. For example, Lee et al. [42] reported that the rainfall-induced soil CO_2_ release accounted for 16–21% of the annual soil respiration in a deciduous forest in Japan, whereas Yuste et al. [43] estimated that approximately 9–14% of the annual soil respiration in Belgian Campine region in Belgium was rainfall induced. The stronger rain pulse responses in grasslands could be the result of more severe water deficiency before rainfall events in grasslands and higher soil organic carbon content at the soil surface layer of grasslands than of shrublands or wood stands [36]. Therefore, the rain pulse effects on belowground C release is critical to ecosystem C balance in the semi-arid steppe ecosystem.

**Figure 5:**
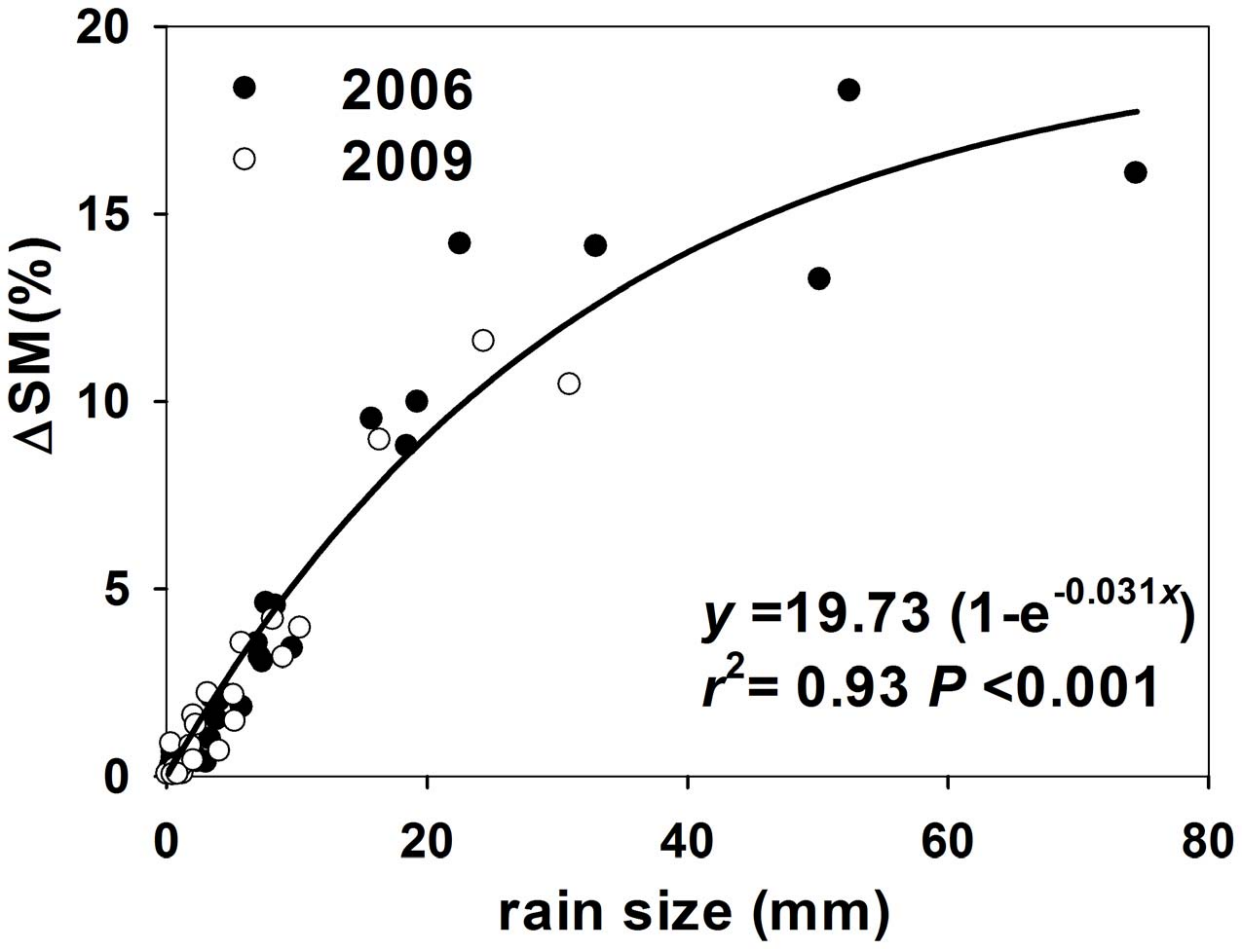
The relationships between the rain size and the change in water availability after a rainfall event (ΔSM; the changes in the largest volumetric soil moisture after a rainfall event from the antecedent volumetric soil moisture) in 2006 (open circle) and 2009 (solid circle) with the coefficient (*r* ^2^) and the significance level (*P*-value).

A rainfall event consists of at least three processes. Firstly, the percolating rainwater replaces the CO_2_ in soil pore spaces. Secondly, the rainwater activates microbial activity and induces microbe-respired CO_2_ in the shallow soil [44]. Thirdly, the rainwater also promotes the assimilation process by roots, which increases root respiration [3], [45]. These processes have been confirmed by the positive relationship found between the rainfall event effect on soil respiration and precipitation in previous studies through field manipulative experiments [4], [7], [18]. Similar trends have also been found in our study. During the two growing seasons of 2006 and 2009, we observed that a larger rainfall event induced a greater pulse effect on soil respiration (Fig. 2a–c). Rather than using the limited water and resources in the soil to maintain activity, most microbes may simply become dormant in the dry soil [46]. The sudden increase in soil water availability after rains can induce microbial cell lysis or the rapid mineralization of cytoplasmic solutes and release the mineralized product into the surrounding environment to dispose of its osmolytes, which have accumulated during the dry period [27], [47]. Consequently, rainfall event induced soil respiration largely comes from the decomposition of microbial cellular material. In addition, more rainfall means more water percolates to the rhizosphere and triggers root activity and respiration [3]. Therefore, the dependence of pulse effect and its duration of soil respiration on precipitation amount in this study can be explained by the greater increase in water availability under heavier rainfall event (Fig. 5).

Although the effects of rainfall events on the changes in water availability before and after rain were similar between 2006 and 2009 (Fig. 5), a significant inter-annual difference in response patterns of soil respiration to rainfall event was found (Fig. 2a–c). It indicates that some other factors rather than rainfall event size regulate the effect of rain pulse on soil respiration. For example, many studies [20], [23], [48] have found that the same amount of water addition had greater effects on soil respiration under drier antecedent soil condition. Soil temperature also has significant effect on soil respiration pulse [5], [24], [36,]. In addition, vegetation, which influences both water and C cycles, are important in regulating the effect of rain pulse on soil respiration [49], [50]. In this study, we introduced the WSI to predict the effect of rain pulse on soil respiration in two growing seasons with contrasting precipitation regimes (Fig. 3d–f). It should be noted that this empirical index does not explain the underlying mechanisms of the response of soil respiration to rainfall events, which have been studied by some hourly-scale stochastic models [37]. However, it highlights the importance of precipitation regime on the pulse response of soil respiration in semi-arid areas. Since the WSI was developed from only two growing-season measurements and based on five chambers, this index needs to be tested in other sites and years before it is used as an indicator for modeling and predicting rain pulse effect on soil respiration in semi-arid grasslands.

### 4.2. Precipitation regime shift and its influence on soil respiration

The long-term precipitation records showed no clear trend of total precipitation amount during a growing season but a higher frequency of heavy rainfall events in our study region (Figure S2 in File S1). It was consistent with the modeling predictions of greater precipitation intensity globally in the future [1], [51]. That is, more precipitation will fall in a given daily rainfall event, leading to more extreme rainfall events in the future. With similar total precipitation amount, higher frequency of heavy rainfall events means longer dry-spells during the growing season (Figure S3 in File S1). Such a shift in precipitation regime has been widely observed [40], [52], [53], although changes in precipitation amount would be greatly different among regions across the globe [2].

We found that a large proportion of the rain pulse effect on soil respiration was determined by the WSI, which considered the influences of rain size and antecedent soil temperature and moisture. The longer dry-spells are likely to lower the antecedent soil moisture. As a consequence, belowground C release is expected to be greater after a rainfall event compared to soils in rainfall regimes with a lower frequency of long dry-spells. The robustness of the WSI in explaining rain pulse effect on soil respiration needs to be examined in other types of grasslands. If the WSI is effective in most grassland ecosystems, it suggests not only total amount of annual precipitation but also the temporal distribution of rainfall events must be taken into consideration when predicting the belowground C release and evaluating C budget of an ecosystem undergoing climate change.

### 4.3. Implications for modeling soil carbon release

Currently, process-based ecosystem models are common tools for simulating and predicting the future states of terrestrial C cycle [2]. Most of these models are established at hourly or daily time scales without incorporating the immediate pulse responses of soil respiration to rainfall events. Therefore, rain pulse effect on soil respiration is one of the key sources of the uncertainty in the modeled ecosystem C cycle, especially in grasslands [8], [36]. In this study, we found that the rain pulse effect on soil respiration and its duration increased with the product of rain size and antecedent soil temperature-to-moisture ratio. However, the relationship between the WSI and rain pulse effect on soil respiration (*y* = 0.1169×WSI) as well as its duration (*y* = 30.13(1-e^−0.021*x*^)) was obtained from the data of only one grassland site during two growing seasons. The parameters in the regression model might vary with soil texture and soil organic matter content, which supplies C substrate for respiration [5], [20], [27]. Therefore, the applicability of the WSI we proposed in this study still needs to be tested in broader spatial scales and at more locations. Parameter information that constrains the relationship between the WSI and rain pulse effect on soil respiration could be useful for improving our ability to predict soil C cycle under future precipitation regimes.

There is evidence that CO_2_ from carbonates may [54] or may not [55] contribute significantly to total CO_2_ release when soils are moistened. Since the soil in our site is calcic, it is still unclear whether rain pulse effect will increase CO_2_ release from inorganic C sources. Future study may pay more attention to the pulse effect of rainfall events on not only the decomposition of soil organic matters but also CO_2_ release from inorganic C sources.

## Conclusions

In this study, we presented a water status index (WSI) as a good indicator for predicting the effect of rain pulse on soil respiration in a temperate steppe in northern China. Long-term precipitation data showed a significant increase in frequency of large rainfall events and dry-spell duration with time. Although there are no measurements of soil temperature and moisture from the historical record to compare against measurements made in 2006 and 2009, the longer dry-spell may leads to larger antecedent ratio between temperature:moisture in the soils, and thus greater rain pulse effect on soil respiration. In this study, rain pulse effect contributed to about 39–44.5% of the growing-season total soil respiration, which is much larger than previous studies in this [4] and other [23] ecosystems. It indicates that the effect of rain pulses on soil respiration cannot be neglected in future climate-carbon cycle modeling. Future research could test the robustness of WSI in other regions before it is used as a simple approach to estimate the rain pulse effect on soil respiration in grassland ecosystems.

## Supporting Information

File S1 **Supporting figures.** **Figure S1**, Half-hourly soil respiration rate (SR, µmol m^−2^ s^−1^) under different small rainfall events. Rainfall size is shown in each panel. The 0 in the x-axis is the beginning time of a rainfall. **Figure S2**, The precipitation amount during the growing season (a) and the occurrence frequency of the nine class-categories of precipitation during the growing season (b–l) from 1953 to 2009. **Figure S3**, The mean dry-spell duration during the growing season (a) and the occurrence frequency of the five class-categories of the dry-spell duration during the growing season (b–f) from 1953 to 2009.
(DOCX)
