# Supplementary material for: Precipitation Regime Shift Enhanced the Rain Pulse Effect on Soil Respiration in a Semi-Arid Steppe
Source: PLoS One. 2014 Aug 5;9(8):e104217. doi: 10.1371/journal.pone.0104217 (PMC4122403; doi:10.1371/journal.pone.0104217)

**Supplementary material**

**Figure S1** Half-hourly soil respiration rate (SR, µmol m^-2^ s^-1^) under different small rainfall events. Rainfall size is shown in each panel. The 0 in the x-axis is the beginning time of a rainfall.


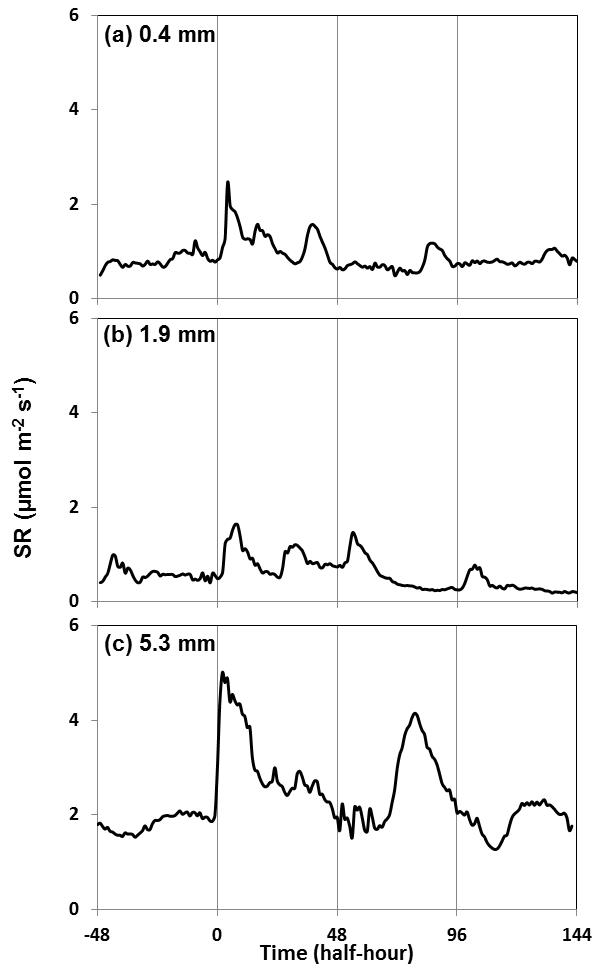


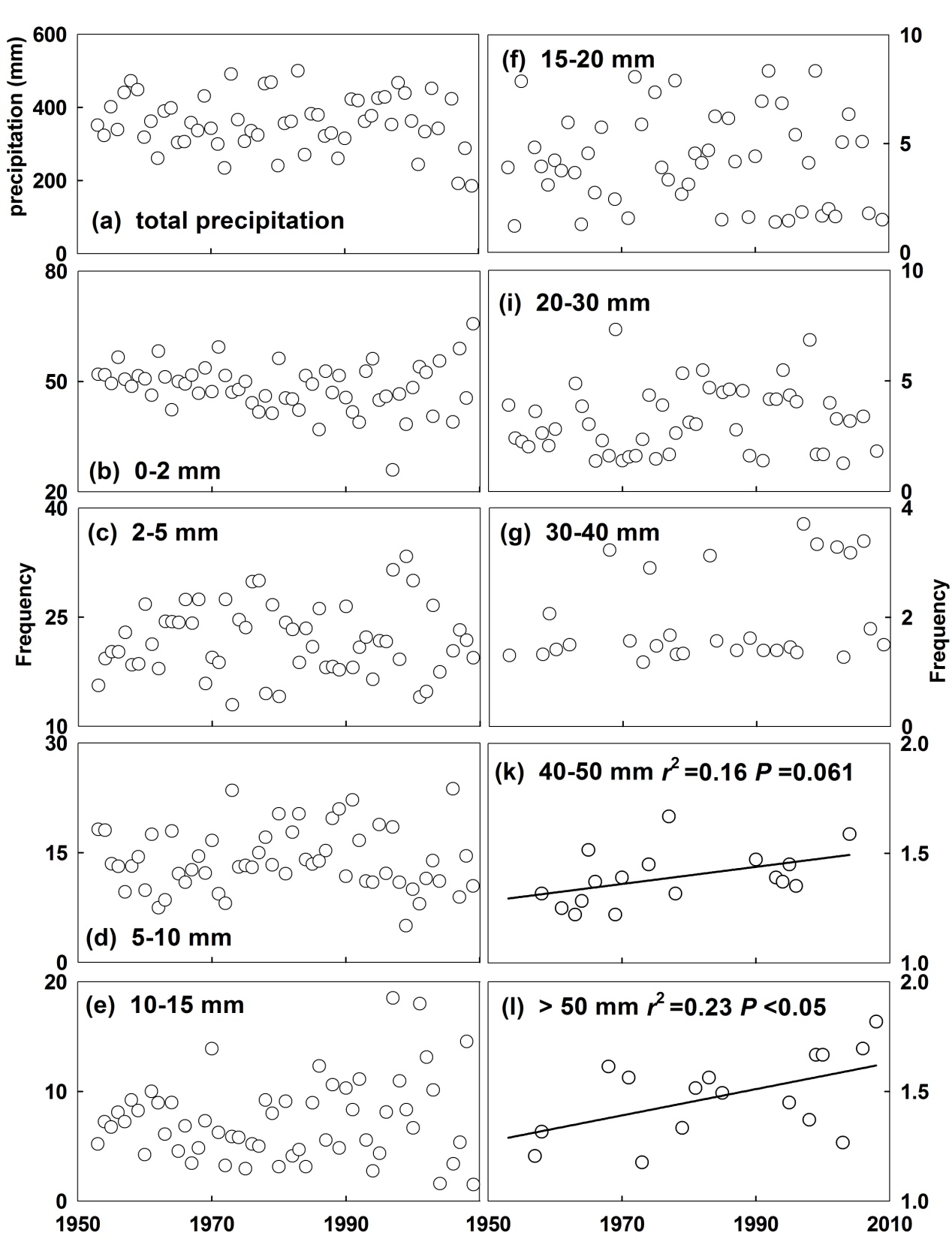
**Figure S2** The precipitation amount during the growing season (a) and the occurrence frequency of the nine class-categories of precipitation during the growing season (b-l) from 1953 to 2009.

**Figure S3** The mean dry-spell duration during the growing season (a) and the occurrence frequency of the five class-categories of the dry-spell duration during the growing season (b-f) from 1953 to 2009.


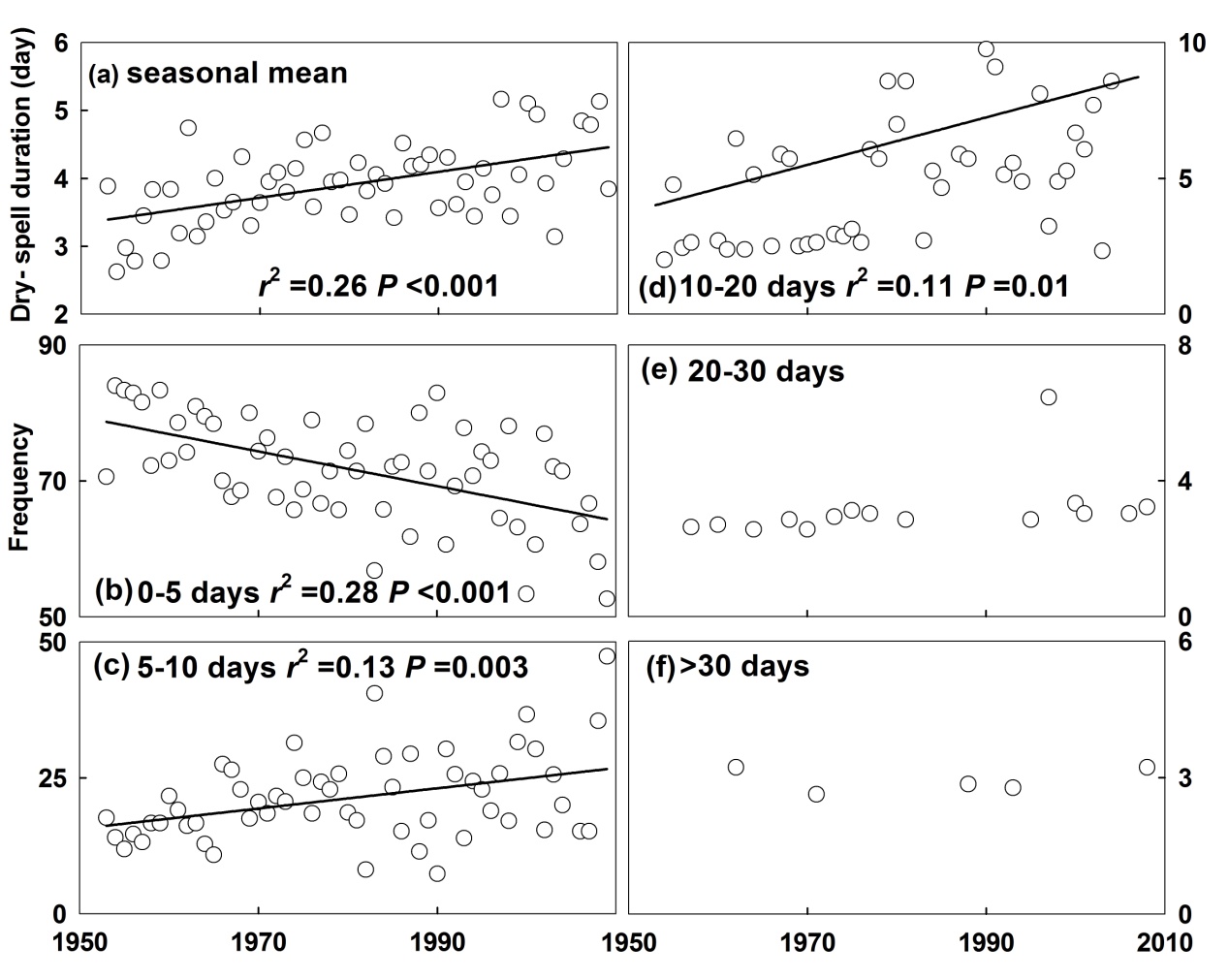

Supplement: File S1 — Supporting figures. Figure S1, Half-hourly soil respiration rate (SR, µmol m−2 s−1) under different small rainfall events. Rainfall size is shown in each panel. The 0 in the x-axis is the beginning time of a rainfall. Figure S2, The precipitation amount during the growing season (a) and the occurrence frequency of the nine class-categories of precipitation during the growing season (b–l) from 1953 to 2009. Figure S3, The mean dry-spell duration during the growing season (a) and the occurrence frequency of the five class-categories of the dry-spell duration during the growing season (b–f) from 1953 to 2009. (DOCX) [file pone.0104217.s001.docx]
